# Torsemida em Comparação com Furosemida no Tratamento da Insuficiência Cardíaca: Uma Revisão Sistemática e Metanálise de Ensaios Clínicos Randomizados

**DOI:** 10.36660/abc.20230825

**Published:** 2024-07-01

**Authors:** Larissa Teixeira, Nicole Felix, Denilsa D. P. Navalha, Rafael Ferreira, Mariana R.C. Clemente, Thiago Madeira, Alleh Nogueira, Lucas Tramujas

**Affiliations:** 1 Universidade Federal de Campina Grande Campina Grande PB Brasil Universidade Federal de Campina Grande, Campina Grande, PB – Brasil; 2 Universidade Eduardo Mondlane Maputo Moçambique Universidade Eduardo Mondlane, Maputo – Moçambique; 3 Universidade Federal de Santa Catarina Florianópolis SC Brasil Universidade Federal de Santa Catarina, Florianópolis, SC – Brasil; 4 Faculdade de Medicina de Petrópolis Petrópolis RJ Brasil Faculdade de Medicina de Petrópolis, Petrópolis, RJ – Brasil; 5 Universidade Federal de Minas Gerais Belo Horizonte MG Brasil Universidade Federal de Minas Gerais, Belo Horizonte, MG – Brasil; 6 Escola Bahiana de Medicina e Saúde Pública Salvador BA Brasil Escola Bahiana de Medicina e Saúde Pública, Salvador, BA – Brasil; 7 Instituto de Pesquisas São Paulo SP Brasil Instituto de Pesquisas, HCor, São Paulo, SP – Brasil

**Keywords:** Insuficiência Cardíaca, Inibidores de Simportadores de Cloreto de Sódio e Potássio, Furosemida

## Abstract

A furosemida é o diurético mais utilizado para o tratamento de sintomas de sobrecarga de volume em pacientes com insuficiência cardíaca. Dados recentes sugerem que a torsemida pode ser superior à furosemida neste contexto. No entanto, ainda não é claro se isso se traduz em melhores resultados clínicos nesta população.

Avaliar se a torsemida é superior à furosemida no contexto da insuficiência cardíaca.

Realizamos uma revisão sistemática e metanálise de estudos clínicos randomizados (ECRs) comparando a eficácia da torsemida em comparação com a furosemida em pacientes com insuficiência cardíaca. PubMed, Embase e Web of Science foram as bases de dados pesquisadas em busca de estudos elegíveis. Os desfechos de interesse foram internações por todas as causas, internações por insuficiência cardíaca (IIC), internações por todas as causas cardiovasculares, mortalidade por todas as causas, e melhoria de classe da NYHA. Parâmetros ecocardiográficos também foram avaliados. Foi aplicado um modelo de efeitos aleatórios para calcular as razões de risco (RR) e as diferenças médias (DM) com intervalos de confiança (IC) de 95% e nível de significância de 0,05.

Foram incluídos 12 ECRs, envolvendo 4.115 pacientes. A torsemida reduziu significativamente a IIC (RR de 0,60; IC de 95%, 0,43-0,83; p=0,002; I^2^=0%), internação por causas cardiovasculares (RR de 0,72; IC de 95%, 0,60-0,88; p=0,0009; I^2^=0%), e melhora da fração de ejeção do ventrículo esquerdo (FEVE) (DM de 4,51%; IC de 95%, 2,94 a 6,07; p<0,0001; I^2^=0%) em comparação com a furosemida. Não houve diferença significativa no número de internações por todas as causas (RR de 0,93; IC de 95%, 0,86-1,00; p=0,04; I^2^=0%), mortalidade por todas as causas (RR de 0,98; IC de 95%, 0,87-1,10; p=0,73; I^2^=0%), melhora da classe NYHA (RR de 1,25; IC de 95%, 0,92-1,68; p=0,15; I^2^=0%), ou mudança de classe NYHA (DM de -0,04; IC de 95%, -0,24 a 0,16; p=0,70; I^2^=15%) entre os grupos.

A torsemida reduziu significativamente as internações por insuficiência cardíaca e causas cardiovasculares, melhorando também a FEVE.

## Introdução

A insuficiência cardíaca é uma condição altamente prevalente, associada a alta morbidade, mortalidade e carga econômica global.^1-3^ A furosemida é o diurético mais comumente usado para aliviar os sintomas de sobrecarga de volume em pacientes com insuficiência cardíaca.^1,4^ No entanto, dados recentes apontam potenciais benefícios da torsemida nesse mesmo contexto, mostrando resultados promissores no alívio sintomático e na redução de internações por insuficiência cardíaca (IIC).^5-10^

Embora a torsemida e a furosemida sejam ambos diuréticos de alça com mecanismos semelhantes, suas propriedades farmacocinéticas distintas podem conferir à torsemida uma maior biodisponibilidade, maior ligação às proteínas e uma meia-vida mais longa.^1,9^ Além disso, estudos demonstraram que a torsemida é capaz de atenuar a remodelação do ventrículo esquerdo (VE) em maior grau do que a furosemida em pacientes com insuficiência cardíaca crônica.^10-13^ No entanto, ainda não está claro se essas diferenças se traduzem em melhores resultados clínicos para essa população de pacientes.

Metanálises anteriores compararam torsemida com furosemida em pacientes com insuficiência cardíaca, produzindo resultados conflitantes. No entanto, tais metanálises incluíram estudos observacionais e dados de curto prazo, o que pode introduzir vieses de seleção e confusão, além de limitar a generalização de seus resultados para o cenário de longo prazo.^5,6,14^ Com isso em mente, nosso objetivo é realizar uma revisão sistemática e metanálise de estudos clínicos randomizados (ECRs) comparando torsemida com furosemida em pacientes com insuficiência cardíaca, buscando avaliar os resultados de eficácia com um período mínimo de acompanhamento de três meses.

**Figure:**
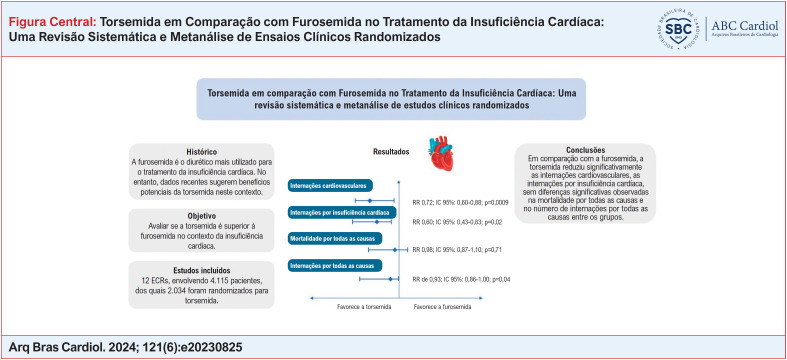


## Métodos

Esta revisão sistemática e metanálise foi conduzida conforme as diretrizes dos *Preferred Items for Systematic Reviews and Meta-analyses* (PRISMA) 2020 e o *Cochrane Handbook of Systematic Reviews of Interventions*.^15,16^ Como tal, seu protocolo foi registrado prospectivamente no Banco de Dados do Registro Prospectivo Internacional para Revisões Sistemáticas (PROSPERO) sob o número de protocolo CRD42023402131.

### Estratégia de pesquisa e extração de dados

Pesquisamos sistematicamente as bases de dados PubMed, Embase e Web of Science desde o início até junho de 2023 usando os seguintes termos de pesquisa: "torsemide", "torasemide" "furosemide", "heart failure", "cardiac failure", "chronic heart failure", "HF", "CHF", "RCT", "random", "randomly", "randomized", "randomization", e "trial". Nenhum filtro ou limitação de idioma foi aplicado à nossa pesquisa. A estratégia de pesquisa exata é exibida na primeira seção do Apêndice Complementar.

Além disso, realizamos uma busca retroativa tipo "bola de neve" para estudos elegíveis adicionais, usando revisões de literatura anteriores, metanálises e estudos incluídos. De forma independente, dois autores (L.T. e D.N.) realizaram a pesquisa e três (L.T., D.N. e M.C.) realizaram a extração dos dados seguindo critérios pré-definidos e avaliação de qualidade. Eventuais conflitos foram resolvidos por consenso.

### Critério de elegibilidade

Restringimos a inclusão de artigos a esta metanálise aos seguintes critérios de elegibilidade: (1) ECRs; (2) comparação de torsemida com furosemida; (3) inclusão de pacientes com insuficiência cardíaca; (4) acompanhamento mínimo de três meses. Foram excluídos: (1) estudos que não relatam qualquer um dos nossos desfechos de interesse; (2) subanálise dos estudos incluídos; e (3) estudos cruzados.

### Endpoints e subanálises

Nossos desfechos clínicos de interesse foram mortalidade por todas as causas, internações por todas as causas, internações por insuficiência cardíaca (IIC), internações cardiovasculares e melhora da classe da New York Heart Association (NYHA). Outros desfechos analisados foram peso corporal, níveis de NT-proBNP, medidas de pressão arterial sistólica (PAS) e pressão arterial diastólica (PAD) e parâmetros ecocardiográficos, como FEVE, índice de massa ventricular esquerda (IMVE) e volume diastólico final do ventrículo esquerdo (VDFVE).

### Avaliação de qualidade e análise de sensibilidade

A qualidade dos ECRs foi avaliada utilizando a ferramenta da Colaboração Cochrane, para avaliar o risco de viés em estudos randomizados (RoB-2), que classifica os estudos em termos de risco de viés alto, baixo ou pouco claro em cinco domínios: seleção, desempenho, detecção, atrito e viés de relatórios.^17^ Além disso, os potenciais efeitos de pequenos estudos (viés de publicação) foram avaliados por meio de análise de gráficos de funil, que mostram a distribuição gráfica de estudos com pesos semelhantes em relação aos seus erros padrão.^18^

Também avaliamos a influência individual dos estudos, removendo sequencialmente cada ECR e reanalisando os dados restantes (análise do tipo "leave-one-out" ou de exclusão). A dominância do estudo foi atribuída ao ECR sempre que os valores p do tamanho do efeito agrupado ao remover o estudo mudaram de significativo para não significativo, ou vice-versa.^19^

### Análise estatística

Usamos o Review Manager 5.4. e R versão 4.2.1 (R Foundation for Statistical Computing, Viena, Áustria) para todas as análises estatísticas.^20,21^ Aplicamos um modelo de efeitos aleatórios de Mantel-Haenszel para agrupamento das razões de risco (RR) com intervalos de confiança (IC) de 95%, um nível de significância de 0,05 para endpoints binários, além de um modelo de efeitos aleatórios de variância inversa para agrupar diferenças médias (DM) com IC de 95% e um nível de significância de 0,05 para dados contínuos para comparar os efeitos dos tratamentos. O teste Cochrane Q e estatística I foram utilizados para avaliar a heterogeneidade entre os estudos; valores de p ≤ 0,10 foram considerados significativos para heterogeneidade.

## Resultados

### Seleção e características dos estudos

**Figura 1:**
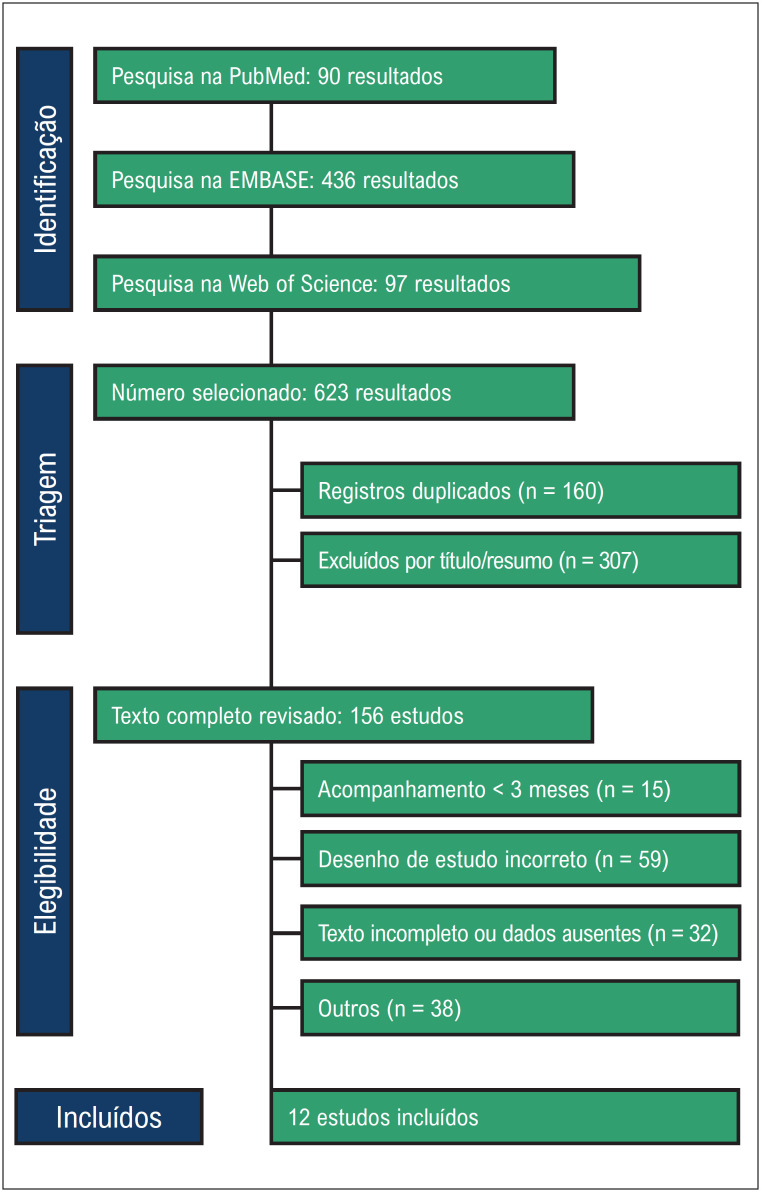
Fluxograma PRISMA de triagem e seleção de estudos.

Conforme ilustrado na Figura 1, nossa pesquisa inicial produziu 623 resultados. Após a remoção dos registros duplicados e a triagem de títulos e resumos, 156 estudos permaneceram elegíveis para revisão de texto completo. Destes, 12 ECRs foram incluídos. A idade média da população agrupada variou de 63 a 75,1 anos. As características individuais do estudo são exibidas na Tabela 1.

**Tabela 1:** Características basais dos estudos incluídos

Características basais		DROP-PIP 2017	KASAMA 2006	LOPEZ 2004	LOPEZ 2007	LOPEZ 2009	MULLER 2003	MURRAY 2001	NOE 1999	STROUPE 2000	TORAFIC 2011	TORNADO 2019	TRANSFORM-HF 2023
**Características do estudo**													
	População do estudo	ICFEp + DM2	ICC	ICC	ICC	ICC	ICC	ICC	ICC	ICC	ICC	IC	ICAD
	Número de pacientes, n (T/F)	35 (17/18)	40 (20/20)	36 (19/17)	22 (11/11)	24 (12/12)	237 (122/115)	234 (113/121)	240 (103/137)	193 (93/100)	155 (77/78)	40 (16/24)	2859 (1431/1428)
	Doses do medicamento (mg) (T/F)	5/20	4-8/20-40‡	10-20/ 20-40‡	10-20/ 20-40‡	10-20/ 20-40‡	10/40	72 ± 76/ 136 ±122*	59/133*	N/A	10/40	70/100*	1:2-4§
	Tempo médio de acompanhamento (meses)	9	6	8	8	8	9	12	6	12	7,3	3	30
**Características dos pacientes**													
	Sexo feminino, n (%)	15 (43)	11 (27,5)	8 (22,2)	5 (22,7)	4 (16,6)	135 (57)	123 (52,6)	107 (44,6)	123 (63,7)	65 (41,9)	9 (22,5)	1055 (37)
	Idade (anos)*	68,7 ± 8,1	68 ± 7,5	63 ± 2,9	64 ± 4,0	66,5 ± 9,3	73,8 ± 10,6	64 ± 11	75,1	63 ± 12	68,7 ± 10,6	66 [51-88]^b^	64,4 ± 14
**Etiologia da IC, n (%)**													
	CPI	NA	0 (0)	9 (25)	5 (22,7)	3 (12,5)	NA	NA	NA	NA	NA	20 (50)	808 (28,2)
	CPH	NA	10 (25)	21 (58,3)	17 (77,3)	17 (70,8)	NA	NA	NA	NA	NA	5 (12,5)	NA
	CPNI†	NA	30 (75)	6 (16,7)	0 (0)	0 (0)	NA	NA	NA	NA	NA	9 (22,5)	NA
**Classe da NYHA, n (%)**													
	I-II	22 (63)	15 (37,5)	13 (36,1)	7 (31,8)	NA	NA	NA	NA	NA	144 (92,9)	NA	NA
	III-IV	13 (37)	25 (62,5)	23 (63,9)	15 (68,2)	NA	NA	NA	NA	NA	11 (7,1)	NA	NA
**Terapia de base para IC, n (%)**			
	Betabloqueador	22 (63)	19 (47,5)	36 (100)	22 (100)	20 (83,3)	NA	48 (20,5)	NA	NA	67 (43,2)	34 (89)	2246 (78,5)
	IECA ou BRA	16 (46)	40 (100)	36 (100)	22 (100)	20 (83,3)	NA	190 (81,2)	NA	NA	75 (48,4)	33 (87)	1243 (43,5)

Esta tabela inclui dados de toda a população do estudo. Foi adotado um nível de significância de 0,05. IECA: inibidor da enzima conversora de angiotensina; ICAD: insuficiência cardíaca aguda descompensada; BRA: bloqueador dos receptores da angiotensina; ICC: insuficiência cardíaca crônica; ICC: insuficiência cardíaca congestiva; CMP: cardiomiopatia; F: furosemida; IC: insuficiência cardíaca; CPH: cardiopatia hipertensiva; ICFEp: insuficiência cardíaca com fração de ejeção preservada; CPI: cardiopatia isquêmica; ARM: antagonistas dos receptores mineralocorticoides; NA: não disponível; CPNI: cardiopatia não isquêmica; NYHA: New York Heart Association; DP: desvio padrão; T: Torsemida; DM2: diabetes mellitus tipo 2.

* Dados expressos em média ou média ± DP.

† Dados expressos em mediana [intervalo interquartil].

‡ Intervalo de doses;

§ Proporção de doses; // Inclui outras etiologias além de DIH e CPH, como cardiomiopatia dilatada ou insuficiência valvar aórtica.

### Análise agrupada dos estudos incluídos

**Figura 2:**
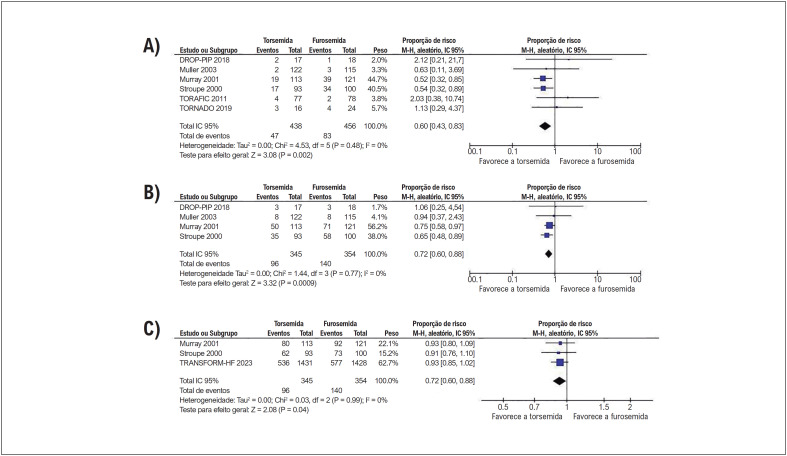
A torsemida reduziu significativamente (A) as IIC e (B) internações cardiovasculares quando comparadas à furosemida. Não houve diferença significativa no número de (C) internações por todas as causas entre os grupos.

Em pacientes com insuficiência cardíaca, a torsemida reduziu significativamente as internações cardiovasculares e IIC em comparação com a furosemida. Não houve diferenças significativas entre os grupos no número de internações por todas as causas (Figura 2).

**Figura 3:**
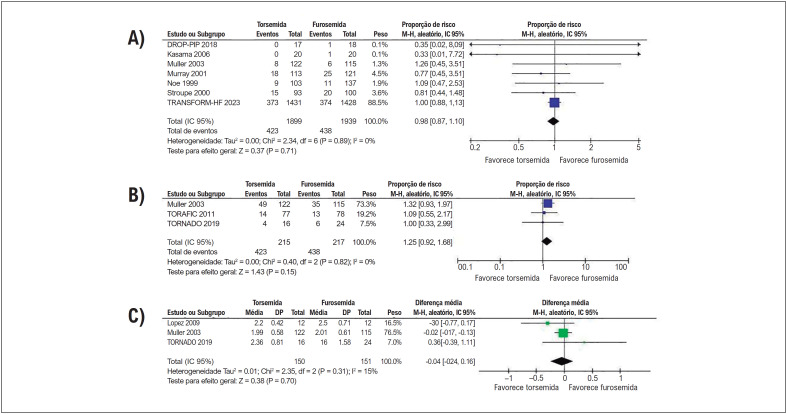
Não houve diferenças significativas entre os grupos em relação a (A) mortalidade por todas as causas, (B) melhora de ≥ 1 classe NYHA, e (C) mudança na classe NYHA.

Não houve diferenças significativas entre os grupos de tratamento em termos de mortalidade por todas as causas, melhora de ≥ 1 classe NYHA, ou mudança da classe basal da NYHA (Figura 3).

**Figura 4:**
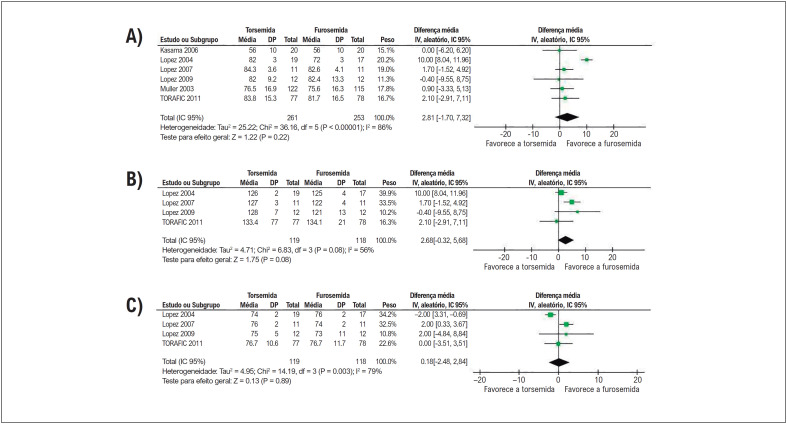
Não houve diferenças significativas entre os grupos em relação a (A) peso corporal, (B) pressão arterial sistólica e (C) pressão arterial diastólica.

Não houve diferença significativa entre os grupos em termos de peso corporal, PAS e PAD (Figura 4).

Quanto aos parâmetros ecocardiográficos, a torsemida melhorou significativamente a FEVE (DM de 4,51%; IC de 95%, 2,94 a 6,07; p<0,001; I^2^=0%; Figura 1a complementar) em comparação com a furosemida. Não houve diferenças entre os grupos no VDFVE (DM de -16,06; IC de 95%, -34,32 a 2,21; p = 0,08; I^2^ = 0%; Figura 1b complementar) ou IMVE (DM de -4,70 g/m^2^; IC de 95%, -10,18 a 0,79; p=0,09; I^2^=9%; Figura 1c complementar).

Não houve diferenças significativas entre os pacientes tratados com torsemida e furosemida em relação aos níveis de NT-proBNP (DM de -226,86 pg/mL; IC de 95%, -443,69 a -10,02; p=0,04; I^2^=0%; Figura 2 complementar).

### Avaliação de qualidade e análise de sensibilidade

**Figura 5:**
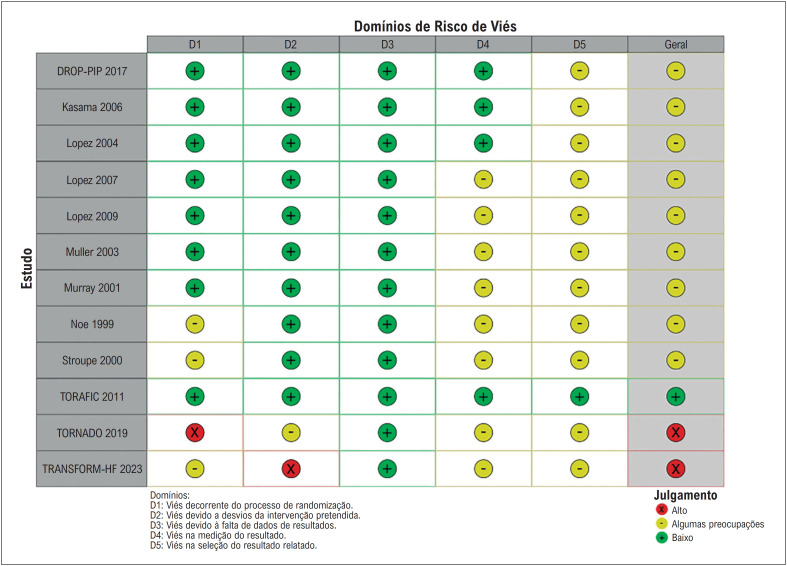
Avaliação do risco de viés para estudos clínicos randomizados.

Dois ECR foram rotulados como de alto risco de viés.^4,22^ Nove foram rotulados como contendo algumas preocupações,^7,8,11,13,23-27^ e um foi rotulado como de baixo risco de viés,^28^ conforme ilustrado na Figura 5. A análise de sensibilidade de exclusão para o desfecho de IIC produziu resultados consistentes, não mostrando dominância do estudo (Figura 3 complementar). A análise do gráfico de funil para o desfecho de IIC não encontrou distribuição assimétrica dos estudos em relação aos seus erros padrão (Figura 4 complementar).

## Discussão

Nesta metanálise de 12 ECRs, comparamos a torsemida com a furosemida em 4.115 pacientes com insuficiência cardíaca. A torsemida foi associada a (1) uma redução de 28% nas internações cardiovasculares, (2) uma redução de 40% nas IIC, e (3) uma melhora na FEVE em comparação com a furosemida. Não foi observada diferença significativa entre os grupos em relação a (4) internações por todas as causas, (5) mortalidade por todas as causas, (6) melhoria da classe NYHA, (7) peso corporal, (8) PAS, (9) PAD, (10) parâmetros ecocardiográficos de IMVE e VDFVE e (11) níveis de NT-proBNP.

Pacientes com insuficiência cardíaca aguda descompensada frequentemente apresentam sintomas de sobrecarga de volume, responsáveis por aproximadamente dois terços das internações hospitalares relacionadas à insuficiência cardíaca e comumente respondem à terapia diurética.^8,28^ As diretrizes atuais de insuficiência cardíaca recomendam diuréticos de alça para o tratamento da retenção de líquidos na dose mais baixa possível para manutenção da euvolemia.^29,30^ Embora a furosemida seja o diurético mais comumente usado na prática clínica, não existem recomendações claras sobre qual diurético de alça deve ser considerado como tratamento de primeira linha.^4,29,30^

Nesse sentido, a torsemida demonstrou melhores características farmacocinéticas e farmacodinâmicas em relação à furosemida em pacientes com insuficiência cardíaca, apesar de seus custos mais elevados.^1,9,10^ Na verdade, ECRs anteriores sugeriram superioridade da torsemida em termos de melhora funcional e social devido à melhor tolerabilidade e diminuição de aspectos de inconveniência, como o número de micções.^7,22,23^ Esses aspectos podem melhorar a adesão do paciente à terapia e podem ser um dos fatores que contribuem para a diminuição das descompensações da insuficiência cardíaca e das IIC,^5-10^ levando assim a uma potencial redução de custos de internação para o sistema de saúde.^10,23^

Nossos resultados mostraram uma redução significativa de internações cardiovasculares e IIC com tratamento com torsemida. Dois mecanismos principais podem contribuir para este achado. Em primeiro lugar, o aumento da biodisponibilidade e uma meia-vida mais longa da torsemida levam a efeitos mais rápidos e mais duradouros, e a uma micção menos frequente em comparação com a furosemida, o que pode conferir uma melhor tolerabilidade global.^1^ Em segundo lugar, a ação da torsemida na ativação neuro-hormonal e seus efeitos antialdosterona atingíveis podem ter impacto na remodelação do VE e nas alterações fibróticas com resultante redução de sintomas, internações e potencial menor mortalidade,^31^ embora nossos achados não tenham conseguido demonstrar uma melhora funcional da NYHA e menor mortalidade em comparação com a furosemida (Figura 3). A torsemida também foi associada a uma melhora na FEVE em comparação com a furosemida, o que é um resultado promissor, especialmente para o subconjunto de pacientes com insuficiência cardíaca com fração de ejeção reduzida (ICFEr).

Metanálises anteriores avaliaram essa comparação. Nossos resultados corroboram os achados de nenhuma diferença significativa na mortalidade por todas as causas e uma taxa significativamente menor de IIC.^5,6,14,31^ No entanto, não encontramos resultados significativos em relação à melhora da classe NYHA, o que diverge em relação aos resultados de estudos anteriores, incluindo estudos observacionais.^5,6^ Em uma metanálise prévia, a melhoria da classe NYHA foi impulsionada por dados observacionais, não significativos no subgrupo de ECR.^6^ Vale ressaltar que duas metanálises recentes foram publicadas sobre este tópico, uma que incluiu ECRs e estudos observacionais^32^ e outra que incluiu apenas ECRs,^33^ também demonstrando uma redução nas IIC e na internação por todas as causas e nenhuma diferença significativa na mortalidade por todas as causas.^32^ Nossa metanálise expandiu os resultados clínicos e avaliou outros parâmetros não previamente agrupados, como resultados ecocardiográficos e laboratoriais. No entanto, resultados publicados conflitantes com estes resultados destacam a necessidade de estudos clínicos adicionais para melhor avaliar esta comparação.

Nosso estudo possui algumas limitações. Em primeiro lugar, apesar de estender o período de acompanhamento para além das metanálises anteriores, a maioria dos estudos incluídos ainda apresentou períodos de acompanhamento relativamente curtos. Em segundo lugar, vários estudos incluídos empregaram desenhos abertos,^4,7,8,22-24^ potencialmente introduzindo vieses tanto dos participantes como dos investigadores. Em terceiro lugar, alguns dos estudos incluídos tinham amostras relativamente pequenas,^7,22,23,25^ o que poderia limitar a precisão das estimativas. No entanto, esta restrição de estudos individuais também destaca a necessidade de combiná-los por meio de uma metanálise para reforçar o poder estatístico das métricas resumidas. Em quarto lugar, a ausência de dados individuais ao nível de paciente nos impediu de realizar subanálises baseadas em fatores que influenciam o endpoint de internação e de realizar análises de subgrupos de acordo com classificações distintas de insuficiência cardíaca. Como as classificações de insuficiência cardíaca variaram substancialmente entre os estudos, não tivemos acesso aos dados individuais dos pacientes ou aos dados agrupados conforme as classes de insuficiência cardíaca, dificultando a avaliação de uma potencial heterogeneidade dos efeitos do tratamento. Por fim, não foi possível realizar uma análise completa dos resultados ecocardiográficos para VDFVE e VSFVE devido aos relatórios incompletos dos estudos individuais.

## Conclusão

Em contraste com os pacientes que receberam furosemida, aqueles com insuficiência cardíaca submetidos à terapia com o diurético torsemida demonstraram melhorias significativas na fração de ejeção do ventrículo esquerdo e reduções no número de internações por insuficiência cardíaca e por causas cardiovasculares. No entanto, não foram identificados impactos discerníveis sobre o número de internações por todas as causas, mortalidade por todas as causas, classe funcional, peso corporal, pressão arterial sistólica e diastólica, níveis de NT-proBNP, índice de massa ventricular esquerda ou volume diastólico final do ventrículo esquerdo. Dada a possibilidade de efeitos clinicamente relevantes para resultados nulos, estudos adicionais serão necessários para atingir uma comparação mais abrangente sobre estes medicamentos.

## *Material suplementar

Para informação adicional, por favor, clique aqui.
