# Supplementary material for: Torsemida em Comparação com Furosemida no Tratamento da Insuficiência Cardíaca: Uma Revisão Sistemática e Metanálise de Ensaios Clínicos Randomizados
Source: Arq Bras Cardiol. 2024 Jul 1;121(6):e20230825. [Article in Portuguese] doi: 10.36660/abc.20230825 (PMC12080595; doi:10.36660/abc.20230825)
Supplement: Translation

# Translation: Torsemide versus Furosemide in the Treatment of Heart Failure: A Systematic Review and Meta-Analysis of Randomized Controlled Trials

**DOI:** 10.36660/abc.20230825i

**Authors:** Larissa Teixeira, Nicole Felix, Denilsa D. P. Navalha, Rafael Ferreira, Mariana R.C. Clemente, Thiago Madeira, Alleh Nogueira, Lucas Tramujas

**Affiliations:** 1 Campina Grande PB Brazil Universidade Federal de Campina Grande, Campina Grande, PB – Brazil; 2 Maputo Mozambique Universidade Eduardo Mondlane, Maputo – Mozambique; 3 Florianópolis SC Brazil Universidade Federal de Santa Catarina, Florianópolis, SC – Brazil; 4 Petrópolis RJ Brasil Faculdade de Medicina de Petrópolis, Petrópolis, RJ – Brasil; 5 Belo Horizonte MG Brazil Universidade Federal de Minas Gerais, Belo Horizonte, MG – Brazil; 6 Salvador BA Brazil Escola Bahiana de Medicina e Saúde Pública, Salvador, BA – Brazil; 7 São Paulo SP Brazil Instituto de Pesquisas, HCor, São Paulo, SP – Brazil

**Keywords:** Heart Failure, Sodium Potassium Chloride Symporter Inhibitors, Furosemide

## Abstract

Furosemide is the most used diuretic for volume overload symptoms in patients with heart failure (HF). Recent data suggested that torsemide may be superior to furosemide in this setting. However, whether this translates into better clinical outcomes in this population remains unclear.

To assess whether torsemide is superior to furosemide in the setting of HF.

We performed a systematic review and meta-analysis of RCTs comparing the efficacy of torsemide versus furosemide in patients with HF. PubMed, Embase, and Web of Science were searched for eligible trials. Outcomes of interest were all-cause hospitalizations, hospitalizations for HF (HHF), hospitalizations for all cardiovascular causes, all-cause mortality, and NYHA class improvement. Echocardiographic parameters were also assessed. We applied a random-effects model to calculate risk ratios (RR) and mean differences (MD) with 95% confidence intervals (CI) and a 0.05 level of significance.

12 RCTs were included, comprising 4,115 patients. Torsemide significantly reduced HHF (RR 0.60; 95% CI, 0.43-0.83; p=0.002; I^2^=0%), hospitalization for cardiovascular causes (RR 0.72; 95% CI, 0.60-0.88; p=0.0009; I^2^=0%), and improved LVEF (MD 4.51%; 95% CI, 2.94 to 6.07; p<0.0001; I^2^=0%) compared with furosemide. There was no significant difference in all-cause hospitalizations (RR 0.93; 95% CI, 0.86-1.00; p=0.04; I^2^=0%), all-cause mortality (RR 0.98; 95% CI, 0.87-1.10; p=0.73; I^2^=0%), NYHA class improvement (RR 1.25; 95% CI, 0.92-1.68; p=0.15; I^2^=0%), or NYHA class change (MD -0.04; 95% CI, -0.24 to 0.16; p=0.70; I^2^=15%) between groups.

Torsemide significantly reduced hospitalizations for HF and cardiovascular causes, also improving LVEF.

## Introduction

Heart failure (HF) is a widely prevalent condition associated with high morbidity, mortality, and economic burden worldwide.^1-3^ Furosemide is the most used diuretic for the relief of volume overload symptoms in patients with HF.^1,4^ Recent data suggested potential benefits of torsemide in this setting, with promising results on symptomatic relief and reduced hospitalizations for heart failure (HHF).^5-10^

Although torsemide and furosemide are loop diuretics with similar mechanisms, their different pharmacokinetic properties may render torsemide with greater bioavailability, a higher degree of protein binding, and a longer half-life.^1,9^ In addition, torsemide has been shown to attenuate left ventricular (LV) remodeling to a greater extent in patients with chronic HF as compared with furosemide.^10-13^ However, whether this translates into better clinical outcomes in this patient population remains unclear.

Previous meta-analyses compared torsemide versus furosemide in patients with HF, yielding conflicting results. Nonetheless, they included observational studies and shorter-term data, potentially introducing selection bias and confounding as well as limiting the generalizability of its results in the long-term setting.^5,6,14^ Herein, we aimed to perform a systematic review and meta-analysis of randomized clinical trials (RCTs) comparing torsemide versus furosemide in patients with HF for efficacy outcomes with a minimum follow-up of three months.

## Methods

This systematic review and meta-analysis was conducted in accordance with the Preferred Items for Systematic Reviews and Meta-analyses (PRISMA) 2020 guidelines and the Cochrane Handbook of Systematic Reviews of Interventions.^15,16^ As such, its protocol was prospectively registered with the International Prospective Register for Systematic Reviews (PROSPERO) database under protocol number CRD42023402131.

### Search strategy and data extraction

We systematically searched PubMed, Embase, and Web of Science from inception to June 2023 using the following search terms: ‘torsemide,’ ‘torasemide,’ ‘furosemide,’ ‘heart failure,’ ‘cardiac failure,’ ‘chronic heart failure,’ ‘HF,’ ‘CHF,’ ‘RCT,’ ‘random,’ ‘randomly,’ ‘randomized,’ ‘randomization,’ & ‘trial.’ No filters or language limitations were applied to our search. The exact search strategy is displayed in the first section of the Supplemental Appendix.

We also performed a backward snowballing search for additional eligible studies using previous literature reviews, meta-analyses, and included studies. Independently, two authors (L.T. and D.N.) performed the search, and three (L.T., D.N., and M.C.) conducted the data extraction following predefined criteria and quality assessment. Eventual conflicts were resolved through consensus.

### Eligibility criteria

We restricted inclusion in this meta-analysis to the following eligibility criteria: (1) RCTs; (2) comparing torsemide with furosemide; (3) enrolling patients with HF; (4) with a minimum follow-up of three months. We excluded (1) studies not reporting any of our outcomes of interest, (2) subanalysis of included trials, and (3) crossover trials.

### Endpoints and sub-analyses

Our clinical outcomes of interest were all-cause mortality, all-cause hospitalizations, hospitalizations for HF (HHF), cardiovascular hospitalizations, and New York Heart Association (NYHA) class improvement. Other outcomes analyzed were body weight, NT-proBNP levels, systolic blood pressure (SBP) and diastolic blood pressure (DBP) measures, and echocardiographic parameters such as left ventricular ejection fraction (LVEF), left ventricular mass index (LVMI) and left ventricular end-diastolic volume (LVEDV).

### Quality assessment and sensitivity analysis

Quality assessment of RCTs was performed using the Cochrane Collaboration’s tool for assessing risk of bias in randomized trials (RoB-2), in which studies are graded as high, low, or unclear risk of bias in five domains: selection, performance, detection, attrition, and reporting biases.^17^ Furthermore, potential small study effects (publication bias) were evaluated through funnel plots analysis of the graphical distribution of studies with similar weights against their standard errors.^18^

We also assessed the individual influence of the studies by sequentially removing each RCT and reanalyzing the remaining data (leave-one-out analysis). Study dominance was assigned to the study whenever pooled effect size p-values when removing the study changed from significant to non-significant, or vice-versa.^19^

### Statistical analysis

We used Review Manager 5.4. and R version 4.2.1 (R Foundation for Statistical Computing, Vienna, Austria) for all statistical analyses.^20,21^ We applied a Mantel-Haenszel random-effects model to pool risk ratios (RR) with 95% confidence intervals (CI) and a 0.05-level of significance for binary endpoints, as well as an inverse variance random-effects model to pool mean differences (MD) with 95% CI and a 0.05-level of significance for continuous data to compare treatments effects. Cochrane Q test and I statistics were used to assess between-study heterogeneity; p-values ≤ 0.10 were considered significant for heterogeneity.

## Results

### Study selection and characteristics

As depicted in Figure 1, our initial search yielded 623 results. After removing duplicate records and conducting title and abstract screening, 156 studies remained eligible for full-text review. Of these, 12 RCTs were included. The pooled population’s average age ranged from 63 to 75.1 years. Individual study characteristics are displayed in Table 1.

**Figure 1:** PRISMA flow diagram of study screening and selection.

**Table 1:** Baseline characteristics of included studies

Baseline characteristics		DROP-PIP 2017	KASAMA 2006	LOPEZ 2004	LOPEZ 2007	LOPEZ 2009	MULLER 2003	MURRAY 2001	NOE 1999	STROUPE 2000	TORAFIC 2011	TORNADO 2019	TRANSFORM-HF 2023
**Study characteristics**													
	Population of study	HFpEF + T2DM	CHF¶	CHF	CHF	CHF	CHF	CHF	CHF¶	CHF¶	CHF	HF	ADHF
	Number of patients, n (T/F)	35 (17/18)	40 (20/20)	36 (19/17)	22 (11/11)	24 (12/12)	237 (122/115)	234 (113/121)	240 (103/137)	193 (93/100)	155 (77/78)	40 (16/24)	2859 (1431/1428)
	Medication doses (mg) (T/F)	5/20	4-8/20-40‡	10-20/ 20-40‡	10-20/ 20-40‡	10-20/ 20-40‡	10/40	72 ± 76/ 136 ±122*	59/133*	N/A	10/40	70/100*	1:2-4§
	Mean time of follow-up (months)	9	6	8	8	8	9	12	6	12	7.3	3	30
**Patient characteristics**													
	Female sex, n (%)	15 (43)	11 (27.5)	8 (22.2)	5 (22.7)	4 (16.6)	135 (57)	123 (52.6)	107 (44.6)	123 (63.7)	65 (41.9)	9 (22.5)	1055 (37)
	Age (years)*	68.7 ± 8.1	68 ± 7.5	63 ± 2.9	64 ± 4.0	66.5 ± 9.3	73.8 ± 10.6	64 ± 11	75.1	63 ± 12	68.7 ± 10.6	66 [51-88]^b^	64.4 ± 14
**HF etiology, n (%)**													
	IHD	NA	0 (0)	9 (25)	5 (22.7)	3 (12.5)	NA	NA	NA	NA	NA	20 (50)	808 (28.2)
	HHD	NA	10 (25)	21 (58.3)	17 (77.3)	17 (70.8)	NA	NA	NA	NA	NA	5 (12.5)	NA
	NIHD†	NA	30 (75)	6 (16.7)	0 (0)	0 (0)	NA	NA	NA	NA	NA	9 (22.5)	NA
**NYHA class, n (%)**													
	I-II	22 (63)	15 (37.5)	13 (36.1)	7 (31.8)	NA	NA	NA	NA	NA	144 (92.9)	NA	NA
	III-IV	13 (37)	25 (62.5)	23 (63.9)	15 (68.2)	NA	NA	NA	NA	NA	11 (7.1)	NA	NA
**Background HF therapy, n (%)**													
	Beta-blocker	22 (63)	19 (47.5)	36 (100)	22 (100)	20 (83.3)	NA	48 (20.5)	NA	NA	67 (43.2)	34 (89)	2246 (78.5)
	ACEi or ARB	16 (46)	40 (100)	36 (100)	22 (100)	20 (83.3)	NA	190 (81.2)	NA	NA	75 (48.4)	33 (87)	1243 (43.5)

This table includes data from the entire study population. A 0.05-level of significance was adopted. ACEi: angiotensin-converting enzyme inhibitor; ADHF: acute decompensated heart failure; ARB: angiotensin receptor blocker; CHF: chronic heart failure; CHF¶: congestive heart failure; CMP: cardiomyopathy; F: furosemide; HF: heart failure; HHD: hypertensive heart disease; HFpEF: heart failure with preserved ejection fraction; IHD: ischemic heart disease; MRA: mineralocorticoid receptor antagonists; NA: not available; NIHD: non-ischemic heart disease; NYHA: New York Heart Association; SD: standard deviation; T: torsemide; T2DM: type 2 diabetes mellitus.

* Data expressed in mean or mean ± SD.

† Data expressed in median [interquartile range].

‡ Range of doses;

§ Dose ratio; // Included etiologies other than IDH and HHD, such as dilated cardiomyopathy or aortic valve insufficiency.

### Pooled analysis of included studies

In patients with HF, torsemide significantly reduced cardiovascular hospitalizations and HHF as compared with furosemide. There were no significant differences between groups in all-cause hospitalizations (Figure 2).

**Figure 2:** Torsemide significantly reduced (A) HHF and (B) cardiovascular hospitalizations when compared to furosemide. There was no significant difference in (C) all-cause hospitalizations between groups.

There were no significant differences between treatment groups in terms of all-cause mortality, improvement of ≥ 1 NYHA class, or change from baseline NYHA class (Figure 3).

**Figure 3:** There were no significant differences between groups regarding (A) all-cause mortality, (B) improvement of ≥ 1 NYHA class, and (C) change in NYHA class.

There was no significant difference between groups in terms of body weight, systolic blood pressure and diastolic blood pressure (Figure 4).

**Figure 4:** There were no significant differences between groups in (A) body weight, (B) systolic blood pressure, and (C) diastolic blood pressure.

As for echocardiographic parameters, torsemide significantly improved LVEF (MD 4.51%; 95% CI, 2.94 to 6.07; p<0.001; I^2^=0%; Supplemental Figure 1a) as compared with furosemide. There were no differences between groups in LVEDV (MD -16.06; 95% CI, -34.32 to 2.21; p=0.08; I^2^=0%; Supplemental Figure 1b) or LVMI (MD -4.70 g/m^2^; 95% CI, -10.18 to 0.79; p=0.09; I^2^=9%; Supplemental Figure 1c).

There were no significant differences between torsemide and furosemide-treated patients with regard to NT-proBNP levels (MD -226.86 pg/mL; 95% CI, -443.69 to -10.02; p=0.04; I^2^=0%; Supplemental Figure 2).

### Quality assessment and sensitivity analysis

Two RCTs were labeled as high risk of bias.^4,22^ Nine were labeled as some concerns,^7,8,11,13,23-27^ and one was labeled as low risk of bias,^28^ as depicted in Figure 5. The leave-one-out sensitivity analysis for the outcome of HHF yielded consistent results, showing no study dominance (Supplemental Figure 3). Funnel plot analysis for the outcome of HHF found no asymmetrical distribution of studies against their standard errors (Supplemental Figure 4).

**Figure 5:** Risk of bias assessment for randomized clinical trials.

## Discussion

In this meta-analysis of 12 RCTs, we compared torsemide with furosemide in 4,115 patients with HF. Torsemide was associated with (1) a 28% reduction in cardiovascular hospitalizations, (2) a 40% reduction in HHF, and (3) an improvement in LVEF as compared with furosemide. No significant difference was observed between groups regarding (4) all-cause hospitalizations, (5) all-cause mortality, (6) NYHA class improvement, (7) body weight, (8) SBP, (9) DBP, (10) echocardiographic parameters of LVMI and LVEDV and (11) NT-proBNP levels.

Patients with acute decompensated HF often present with volume overload symptoms, responsible for approximately two-thirds of HF-related hospital admissions and commonly responsive to diuretic therapy.^8,28^ Current HF guidelines recommend loop diuretics for the treatment of fluid retention at the lowest dose possible to maintain euvolemia.^29,30^ Even though furosemide is the most commonly used diuretic in clinical practice, there are no clear recommendations regarding which loop diuretic should be considered first-line.^4,29,30^

In this sense, torsemide has shown better pharmacokinetic and pharmacodynamic features relative to furosemide in patients with HF, albeit with higher costs.^1,9,10^ In fact, previous RCTs suggested the superiority of torsemide in terms of functional and social improvement due to overall better tolerability. They also decreased inconvenient aspects such as a number of mictions.^7,22,23^ These aspects could improve patient compliance with therapy and may be one of the factors contributing to a decrease in HF decompensations and HHF,^5-10^ thus leading to potential inpatient cost savings to the healthcare system.^10,23^

Our results showed a significant reduction in cardiovascular hospitalizations and HHF with torsemide treatment. Two main mechanisms may contribute to this finding. First, increased bioavailability and longer half-life of torsemide lead to faster, longer effects and less frequent micturition compared to furosemide, which may confer overall better tolerability.^1^ Second, torsemide’s action on neurohormonal activation and its attainable anti-aldosterone effects may impact LV remodeling and fibrotic changes with resultant reduced symptoms, hospitalizations and potential lower mortality,^31^ even though our findings failed to demonstrate a NYHA functional improvement and lower mortality compared with furosemide (Figure 3). Torsemide was also associated with an improvement in LVEF compared to furosemide, which is a promising result, especially for the subset of patients with heart failure with reduced ejection fraction (HFrEF).

Previous meta-analyses have assessed this comparison. Our results support findings of no significant difference in all-cause mortality and a significantly lower rate of HHF.^5,6,14,31^ However, we did not find significant results regarding NYHA class improvement, diverging from previous study results that included observational studies.^5,6^ In one meta-analysis, NYHA class improvement was driven by observational data, which was not significant in the RCT subgroup. published on this topic, one which included RCTs and observational studies^32^ and another which included only RCTs,^33^ also demonstrated a reduction in HHF and all-cause hospitalization and no significant difference in all-cause mortality.^32^ Our meta-analysis expanded clinical outcomes and assessed other parameters not previously pooled, such as echocardiographic and laboratory outcomes. Nonetheless, conflicting published results on these outcomes highlight the need for additional clinical trials to assess this comparison further.

Our study possesses limitations. First, despite extending the follow-up period beyond previous meta-analyses, most of the studies we included still had relatively short follow-up durations. Second, a number of the included studies employed open-label designs,^4,7,8,22-24^ potentially introducing biases from both participants and investigators. Third, some of the included studies had relatively small sample sizes,^7,22,23,25^ which could limit the precision of estimates. However, this constraint of individual studies also underscores the necessity of combining them through meta-analysis to bolster the statistical power of summary metrics. Fourth, the absence of individual patient-level data prevented us from performing subanalyses based on factors influencing the hospitalization endpoint and conducting subgroup analyses according to distinct HF classifications. As HF classifications varied substantially across the studies, we lacked access to individual patient data or grouped data according to HF classes, making it challenging to assess the potential heterogeneity of treatment effects. Finally, we could not conduct a thorough analysis of echocardiographic outcomes for LVEDV and LVESV due to incomplete reporting in the individual studies.

## Conclusion

In contrast to patients receiving furosemide, those with heart failure undergoing torsemide diuretic therapy demonstrated significant enhancements in left ventricular ejection fraction and reductions in hospitalizations for heart failure and cardiovascular causes. However, there were no discernible impacts on all-cause hospitalizations, all-cause mortality, functional class, body weight, systolic and diastolic blood pressure, NT-proBNP levels, left ventricular mass index, or left ventricular end-diastolic volume. Given the possibility of clinically relevant effects for null outcomes, additional trials are necessary to conduct a more comprehensive comparison of these medications.

## *Supplemental Materials

For additional information, please click here
